# Mechanisms of Broad-Spectrum Antiemetic Efficacy of Cannabinoids against Chemotherapy-Induced Acute and Delayed Vomiting

**DOI:** 10.3390/ph3092930

**Published:** 2010-09-03

**Authors:** Nissar A. Darmani

**Affiliations:** Department of Basic Medical Sciences, College of Osteopathic Medicine of the Pacific, Western University of Health Sciences, Pomona, CA, USA; E-Mail: ndarmani@westernu.edu; Tel.: +1-909-469-5654; Fax: +1-909-469-5654

**Keywords:** cannabinoids, acute, delayed, emesis, CB_1_ receptor, endocannabinoids, antiemetics, chemotherapy

## Abstract

Chemotherapy-induced nausea and vomiting (CINV) is a complex pathophysiological condition and consists of two phases. The conventional CINV neurotransmitter hypothesis suggests that the immediate phase is mainly due to release of serotonin (5-HT) from the enterochromaffin cells in the gastrointestinal tract (GIT), while the delayed phase is a consequence of release of substance P (SP) in the brainstem. However, more recent findings argue against this simplistic neurotransmitter and anatomical view of CINV. Revision of the hypothesis advocates a more complex, differential and overlapping involvement of several emetic neurotransmitters/modulators (e.g. dopamine, serotonin, substance P, prostaglandins and related arachidonic acid derived metabolites) in both phases of emesis occurring concomitantly in the brainstem and in the GIT enteric nervous system (ENS) [1]. No single antiemetic is currently available to completely prevent both phases of CINV. The standard antiemetic regimens include a 5-HT_3_ antagonist plus dexamethasone for the prevention of acute emetic phase, combined with an NK_1_ receptor antagonist (e.g. aprepitant) for the delayed phase. Although NK_1_ antagonists behave in animals as broad-spectrum antiemetics against different emetogens including cisplatin-induced acute and delayed vomiting, by themselves they are not very effective against CINV in cancer patients. Cannabinoids such as Δ^9^-THC also behave as broad-spectrum antiemetics against diverse emetic stimuli as well as being effective against both phases of CINV in animals and patients. Potential side effects may limit the clinical utility of direct-acting cannabinoid agonists which could be avoided by the use of corresponding indirect-acting agonists. Cannabinoids (both phyto-derived and synthetic) behave as agonist antiemetics via the activation of cannabinoid CB_1_ receptors in both the brainstem and the ENS emetic loci. An endocannabinoid antiemetic tone may exist since inverse CB_1_ agonists (but not the corresponding silent antagonists) cause nausea and vomiting.

## 1. Introduction

While marijuana is the popular name for the *Cannabis sativa*, cannabis refers to the products of this plant. Cannabis has been used throughout human history for its psychotropic effects (changes in sensory perception, elation and euphoria) and medicinal properties (such as relief of pain, nausea and vomiting). The clinical potential of Δ^9^-THC {(-)-trans-delta-9-tetrahydrocannabinol} and four other cannabinoids (Δ^8^-THC, nabilone, levonantradol and nonabine) against chemotherapy-induced nausea and vomiting (CINV) has been recognized for several decades [2,3]. However, the molecular mechanisms by which these agents prevent emesis [4,5,6,7] were only recently ascertained from animal models of emesis, following the discovery of cannabinoid CB_1_ and CB_2_ receptors [8]. Endogenous ligands (endocannabinoids) for these receptors have also been identified in various tissues including emesis-relevant loci such as the gut and the brainstem. These recent findings advocate that phyto- and synthetic cannabinoids possess broad-spectrum antiemetic properties [2,3]. On the other hand, while endocannabinoids possess limited antiemetic efficacy [6,9,10], some also induce emesis by themselves or potentiate vomiting caused by other agents [11]. Deciphering the role of neurotransmitters in the anatomical substrates through which the sensation of nausea is generated and reflex emetic circuits are activated is cardinal not only to further understanding of the molecular mechanisms of cannabinoids antiemetic actions, but also in gaining new insights into the multifaceted puzzles of the immediate and delayed phases of CINV [1]. Thus, in order to understand how phyto- and synthetic cannabinoids act as broad-spectrum antiemetics, whereas endocannabinoids behave as pro- and/or anti-emetic agents, it is necessary to review: (1) the anatomical and neurotransmitter bases of emesis in general and of chemotherapy-induced acute and delayed phase vomiting in particular; (2) the evidence for cannabinoid biomarkers found in the anatomical substrates of CINV, in both the GIT and the brainstem; and (3) the molecular bases of antiemetic mechanisms of cannabinoids during acute and delayed phase CINV .

## 2. CINV Emetic Circuits

**Figure 1:**
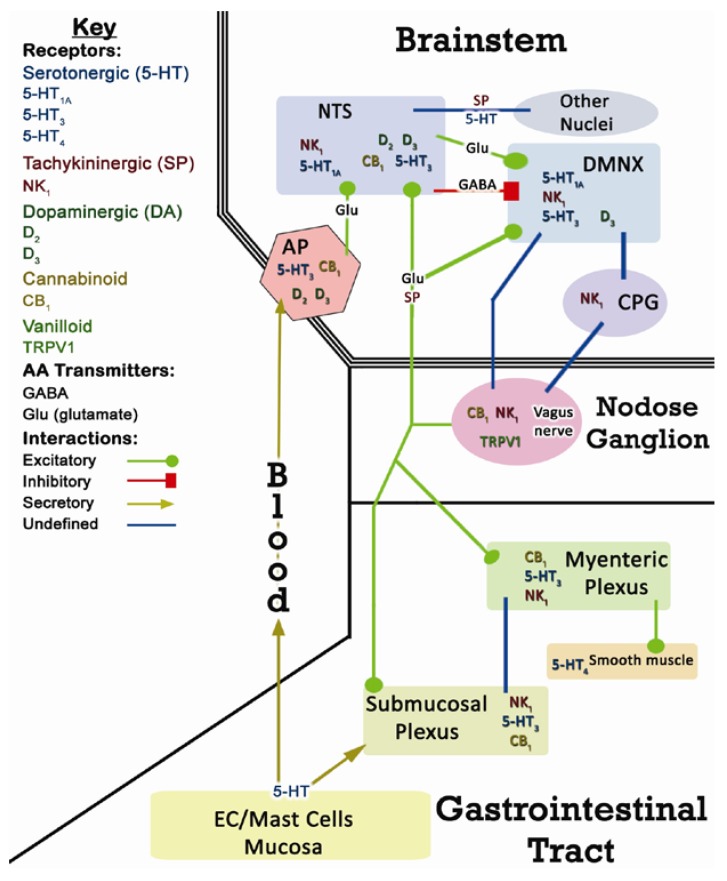
Overview of neurotransmitter systems involved in the emetic reflex. Numerous receptors are present in emetic loci, but only well-established receptor localizations are depicted. The cholinergic system has also been omitted since it does not play a role in mediating CINV. Many connections in the emetic reflex [e.g. the central pattern generator (CPG)] are not well-defined anatomically and/or physiologically (blue lines). EC – intestinal enterochromaffin cells.

The emetic reflex arc is a highly complex system, especially with regard to CINV which is only partially characterized. CINV involves both central and peripheral mechanisms. A simplified overview is illustrated in Figure 1 [1,12,13].

### 2.1. Terminology

Nausea and vomiting are among common complaints when patients visit their physicians. These symptoms may occur separately or together and can result from diverse conditions ranging from gastrointestinal disorders to brain tumors, poisoning, or exposure to drugs. Emesis (also known as vomiting) is a reflex that is present to varying degrees in different species and involves forceful expulsion of the contents of the stomach through the mouth. The continuous feeling of gastrointestinal discomfort that one is about to vomit is called nausea. Often nausea precedes the act of vomiting, however, nausea does not always lead to emesis. This nauseous subjective sensory experience frequently involves disturbed gastrointestinal motility [14]. The act of vomiting is usually preceded by retchings, where the GIT contents are forced into the esophagus, but the vomitus does not enter the pharynx and thus nothing is expelled. On occasions emesis is a beneficial defense mechanism through which the body rids itself of ingested toxins. Conversely, in other cases vomiting can be a disadvantage, .e.g. severe loss of fluid and ion imbalance following exposure to chemotherapeutics such as cisplatin, which could lead to refusal of further therapy by cancer patients. Cisplatin and related drugs produce vomiting biphasically in both humans [14] and other vomiting species [15,16,17,18,19]. In patients, the acute (immediate) emetic phase is comprised of episodes occurring within 24 hours of cisplatin infusion, followed by a quiescent phase with little or no emetic activity, while in the delayed phase, bouts of vomiting continue from days 3-7 post-infusion. A close examination of the published studies in most animal models of CINV shows a similar pattern of vomiting activity. However, the details of temporal development of cisplatin-induced emetic behaviors in animals are shorter and dependent upon the: 1) dose used, 2) route of administration employed, 3) presentation of attained emetic parameters, either as a single parameter or combinations of behaviors, and 4) species used and differences in cisplatin action and disposition among species [15,16,17,18,19,20,21,22].

### 2.2. Peripheral Components of CINV

In CINV and in several other gastrointestinal disorders that manifest vomiting, the emetic signals often initiate in the GIT. Critical to the emetic reflex, enterochromaffin cells (EC) are epithelial cells that act as sentinel cells in the intestinal mucosa. They store serotonin (5-HT) and substance P (SP) as well as other emetogenic proinflammatory mediators [1]. Control of release of such stored transmitters is complex since multiple receptors are present on EC cells that modulate their release. For example, release of 5-HT can be increased via stimulation of serotonergic 5-HT_3_, dopaminergic D_2_, adrenergic β-, cholinergic M_3_-, and nicotinic-receptors, while activation of other receptors inhibits its release (e.g. tachykininergic NK_1_- and NK_3_-, adrenergic α_2_-, purinergic P2Y-, and histaminergic H_3_-receptors) [23,24]. Interestingly, EC cells (or mast cells) can also be stimulated to release 5-HT by prostanoids and CINV-inducing chemicals such as cisplatin [25,26]. Emetogenic neurotransmitters such as dopamine (DA), 5-HT, SP and prostaglandins that are released by cisplatin into the intestinal wall or into the bloodstream can act: (1) directly on corresponding specific receptors present in the enteric nervous system (ENS) plexi and on intestinal smooth muscle to locally modulate intestinal contractility, rhythmicity, retroperistaltic activity, and secretory activity during CINV [27,28,29]; and (2) indirectly to induce vomiting via activation of the CNS emetic nuclei following stimulation of corresponding peripheral receptors present on vagal afferents in the GIT whose somata are in the nodose ganglion and whose terminals are in the area postrema (AP), the nucleus of the solitary tract (NTS), and the dorsal motor nucleus of the vagus nerve (DMNX) within the brainstem. In fact serotonin, via 5-HT_3_ receptors, and SP, via NK_1_ receptors, increase the activity of vagal afferents [30,31]. Other proemetic signals such as prostanoids have also been found to increase excitability of vagal afferent neurons [32,33,34]. Indeed, while no prostaglandin receptors have been identified on confirmed emesis-related vagal afferents, immunolabeling for EP prostaglandin E_2_ receptors (PGE_2_ receptors) has been found in nodose ganglionic neurons [35,36]. Although dopamine D_2_ receptors and mRNA markers for DA synthetic enzymes are present in emesis related nodose ganglionic vagal afferents [37,38], DA or D_2_ selective agonists seems to indirectly increase the spontaneous activity of vagal afferents in the GIT via an increase in 5-HT turnover in the ileum [38,39]. The discussed data strongly suggest that vagal afferents can be an important bridge for the transfer of emetic signals beween the peripheral and central anatomical substrates of CINV.

### 2.3. Central Components of CINV

The discussed brainstem emetic nuclei (AP, NTS and DMNX) are collectively described as the dorsal vagal complex (DVC) and are involved in the central mediation of emesis. Some emetics can pass the blood-brain barrier (BBB) and directly activate the DVC [40]. In fact the AP in the chemoreceptive trigger zone (CTZ) has both fenestrated capillaries and active transport systems which allow bloodborne chemicals absorbed by or secreted from the intestinal mucosa (e.g. SP) to bypass the BBB and directly stimulate the DVC to induce vomiting [12,40]. The AP/CTZ is populated by neurons containing a broad spectrum of emetic receptors, including dopaminergic D_2_-, serotonergic 5-HT_3_ -, tachykinergic NK_1_-, and cholinergic- receptors, resulting in sensitivity to a wide range of chemical signals [1]. The NTS, specifically the medial subnucleus (mNTS), is a point of convergence and is the key integrative site for CNS modulation of the emetic reflex. It receives input from diverse brain nuclei including the vestibular nuclei, vagal afferents, posterior and paraventricular hypothalamic nuclei, the serotonergic raphe nuclei and the other DVC nuclei. As with the AP, the discussed diverse emetogenic receptors are also present in the NTS [20,36,41,42,43,44,45,46,47,48,49]. After integrating the central and peripheral signals relating to emesis or other GI activity, inhibitory GABAergic and excitatory glutamatergic primary NTS neurons project to neurons in the DMNX and to the central pattern generator area (CPG) [40,50]. The DMNX also receives afferents from vagal nodose ganglion neurons, and sends efferents to the enteric nervous system (ENS), as well as to the emetic CPG postulated to be dorsomedial to the nucleus ambiguus and retrofacial nucleus which coordinate peristaltic activity and its reversal during emesis [51,52,53,54]. Electrophysiological findings suggest that vagal afferents innervate the DVC, while axonal branches of these afferents then turn ventrolaterally to innervate the CPG [54]. The DMNX is also endowed with emetic D_2_-, 5-HT_3_- and NK_1_-receptors [1]. Cisplatin-induced vomiting appears to involve stimulation of the entire DVC and the CPG by vagal afferents, which produces the initial giant retroperistaltic contraction to force intestinal contents back to the stomach. Stimulation of the NTS by the AP and vagal afferents then inhibits DMNX motor neuron activity, while exciting the CPG into a shift to burst-firing mode and producing periodic visceral muscle contractions. The effect is to relax the lower esophageal sphincter (LES) muscle, and allow the lower GI and stomach muscles to contract and expel the toxic contents [52,54,55,56].

## 3. Cannabinoids and Endocannabinoids

In addition to the naturally occurring endocannabinoids and phytocannabinoids, numerous direct- and indirect-acting compounds with cannabimimetic activity have been synthesized. The mechanisms by which Δ^9^-THC and its structural analogs produce their cellular effects were revealed after the identification and cloning of at least two G-protein coupled receptors called cannabinoid CB_1_ and CB_2_ [57,58]. Furthermore, physical and genetic localization of cannabinoid receptor genes, CNR1 and CNR2, have been mapped on chromosomes 6 and 1, respectively. While the CB_1_ receptor is expressed in the neurons in the CNS, the CB_2_ receptor is often localized in lymphoid tissues in the periphery. The presence and function of CB_2_ receptors in brain neurons is controversial, although recent evidence suggests their presence on peripheral neurons. Thus far only a few studies indicate the presence of CB_2_-immunoreactivity (IR) or its mRNA expression in the neurons in the brain DVC subnuclei [59]. Δ^9^-THC and other well studied cannabinoids (CP99,994; HU-210; WIN55,212-2) have similar affinities for the two receptors [58]. In more recent years a number of selective CB_1_ agonists(e.g. methanandamide, O-1812) and antagonists (e.g. SR141716A, AM251, AM281) have been synthesized. Selective CB_2_ agonists (e.g. JWH133, AM1241) and antagonists (e.g. SR144528, AM630) have also been discovered. To date at least two well-investigated endocannabinoids are recognized, *N*-arachidonoylethanolamide (also called anandamide) and 2-arachidonoylglycerol (2-AG), in both the brain and the gut. Several pathways exist for their formation and catabolism that are described elsewhere in this volume. Following their cellular reuptake, anandamide is metabolized via fatty acid amide hydrolase (FAAH), and 2-AG via monoacylglycerol lipase (MAGL). 2-AG is also metabolized to some extent by other hydrolases, as well as by FAAH [57]. One major metabolite of both endocannabinoids is arachidonic acid which can be further catabolized to numerous compounds including prostaglandins and leukotrienes, as well as other proinflammatory agents. Several of these prostaglandins and leukotrienes, as well as arachidonic acid itself, are potent emetogens [2,60]. Thus far, only selective inhibitors of FAAH (e.g. URB-597, arachidonoylserotonin, SA7) have been developed which act as indirect agonists and thus can produce cannabimimetic activity. Likewise, selective inhibitors of the endocannabinoid reuptake process are being developed (including OMDM-1 and UCM-707), which also act as indirect agonists. If the latter agents were clinically useful, this would help to avoid the psychoactivity of Δ^9^-THC and its structural analogs. 

Anandamide also interacts with several non-cannabinoid receptors, including the transient receptor potential vanilloid subtype 1 (TRPV1) receptor, to which it binds at an intracellular site. Moreover, both 2-AG and anandamide activate an orphan G-protein-coupled receptor, GPR5 [57]. However, most often the effects of cannabinoids have been studied through CB_1_ and CB_2_ molecular targets. Anandamide has the highest affinity for cannabinoid CB_1_ and CB_2_ receptors, whereas 2-AG has the greatest efficacy. While most clinically useful antiemetics are antagonists of emetic receptors, phytocannabinoids as well as synthetic cannabinoids act as agonist antiemetics via the activation of cannabinoid CB_1_ receptors, whereas endocannabinoids possess both pro- and antiemetic actions [1,2]. Most published studies exclude a role for CB_2_ receptors in emesis, although a recent study indicates a minor role for this receptor in vomiting [59]. Anandamide may also provide protection against emesis via its endovanilloid agonist activity through the activation of TRPV1 receptors [9].

## 4. Cannabinoid Targets in Emetic Circuits

A multitude of experimental findings including anatomical, immunohistochemical, functional and tissue analysis indicate that both cannabinoid CB_1_ and CB_2_ receptors, as well as TRPV1 receptors, and their endogenous ligands, are found in the brainstem/GIT circuits that can affect GIT motility, secretion and function [61,62,63] which would ultimately affect emesis as described below.

### 4.1. Dorsal Vagal Complex (DVC)

Both anandamide and 2-AG are found in significant concentrations in different parts of the mammalian brain. 2-AG tissue levels are approximately one order of magnitude greater than anandamide, with particularly high levels of both endocannabinoids in the brainstem [59,64]. The NTS contains significant concentrations of anandamide [65], but the tissue levels of these endocannabinoids in other subnuclei of the DVC remain to be determined. Immunohistochemical studies show dense levels of CB_1_-immunoreactivity (IR) in the ferret mNTS and DMNX and moderate staining in the area postrema [6,54]. Furthermore, CB_1_-IR terminals surrounded FAAH immunoreactive cell bodies in the ferret DMNX. Immunohistochemical, autoradiographic, and brain homogenate radioligand- and GTPγS-binding studies also indicate a similar distribution of CB_1_ receptors in the least shrew DVC, with CB_1_ receptors being especially dense in its NTS with more sparse levels in the DMNX and AP regions [5,66]. Some punctate CB_1_-IR (putative terminal) labeling in the least shrew was co-localized with punctate immunoreactivity for 5-HT and/or SP neuronal terminals in the NTS [5]. CB_1_-IR and/or mRNA expression is also found in the brainstem subnuclei of other species including humans [67,68]. Cannabinoids may affect several possible sites in the brainstem to reduce chemotherapy-induced vomiting by: (i) acting at presynaptic CB_1_ receptors to inhibit neurotransmitter release from the vagal afferent terminals, thus preventing afferent transmission. Thus, a reduction in Fos-IR would be expected in neurons downstream of these synaptic connections. In fact, this is the case since Δ^9^-THC reduces cisplatin-induced Fos-IR during acute emesis in both the ferret and least shrew NTS and DMNX in a CB_1_ antagonist-sensitive manner [5,57]; (ii) acting on CB_1_ receptors present on the terminals of inhibitory interneurons within the NTS that receive inputs from vagal afferents. These inhibitory interneurons probably reduce the activity of excitatory NTS neurons that project to the DMNX, which could lead to suppression of visceral motor responses [7,69]; (iii) acting on CB_1_ receptors present on the terminals of NTS neurons which project to the DMNX or the AP. As a matter of fact the enhanced Fos activity in both the ferret and least shrew NTS following acute cisplatin-induced vomiting was reduced by Δ^9^-THC in a CB_1_ receptor-dependent fashion [5,7]. The downstream target of this activation, the DMNX, also exhibited decreased Fos activity following Δ^9^-THC pretreatment; and (iv) acting on CB_1_ receptors in the AP which project to the NTS and DMNX.

The large reduction in Fos-IR in the area postrema of cisplatin exposed ferrets and least shrews following Δ^9^-THC pretreatment is probably due to either a modulatory input to the AP from the NTS regulated by CB_1_ receptors, and/or Δ^9^-THC-induced reductions in the release of bloodborne emetogens such as prostaglandins, 5-HT, or SP. 

Labeling for CB_2_ was practically nonexistent in the DVC of the least shrew with the exception of one or two elements morphologically indicative of vascular walls. In addition, the choroid plexus and the surface of the brainstem exhibited moderate levels of CB_2_ immunoreactivity. However, both the ferret and rat AP and DMNX appear to express CB_2_ mRNA, and CB_2_-IR was shown to also occur in the ferret DMNX [59]. Using anandamide and 2-AG as well as indirect agonists (uptake inhibitors or catabolic inhibitors) combined with selective CB_1/2_ antagonists, the latter authors have indicated that CB_2_ receptor activation may also have an antiemetic role against morphine-6-glucuronide-induced vomiting [59]. However, not only the direct-acting and selective synthetic CB_2_ agonists (AM1241 or JWH 133) failed to block the induced emesis in the latter study, previous publications of these authors [6,7,9] and numerous other studies do not support an antiemetic role for CB_2_ receptors against diverse emetogens.

The endovanilloid TRPV1 receptor-IR in the ferret brainstem also appears to be most abundant in the NTS, with less labeling in the DMNX and AP [9]. Within the NTS, TRPV1 receptors were most abundant in the subnucleus gelatinosus, the medial subnucleus and in the solitary tract itself, with labeling mostly localized to fibers and terminals. In addition, a high degree of colocalization of CB_1_ and TRPV1 receptors has been demonstrated in dorsal and medial nuclei of the NTS and in motor neurons of the DMNX, and in a few scattered neurons of the AP. Such colocalization may have functional importance in the antiemetic efficacy of hybrid agonists (e.g. arvanil) stimulating both receptors. Resiniferatoxin obtained from *Euphorbia* sp., is an ultrapotent agonist of TRPV1 receptors. It is an analog of the sensory neurotoxin capsaicin which itself is the hot ingredient of chili peppers. The mechanism and site of antiemetic action of resiniferatoxin has been suggested to be stimulation of TRPV1 receptors in the terminal portion of capsaicin-sensitive, SP-containing emetic vagal afferents in the mNTS. SP is postulated to be the emetic neurotransmitter in the synapse between these vagal afferent terminals and the neurons of the mNTS which drive the CPG to induce emesis [70].

### 4.2. Vagal Afferents

Cannabinoid CB_1_-IR is found on the cell bodies of vagal afferent neurons in the ferret, rat and human nodose ganglion, and CB_1_ receptor is largely transported to the peripheral terminals rather than to central terminals [54,71]. Not only can cannabinoids affect emesis through modulation of vagal afferent activity to the DVC nuclei, but they can also act via vagal efferents, since gastric motor inhibition caused by systemic Δ^9^-THC can be abolished by vagotomy, and Δ^9^-THC applied to the dorsal surface of the medulla mimics the effect of intravenously-administered Δ^9^-THC [72]. Vagal efferents have their cell bodies in the DMNX and project to both submucosal and myenteric plexi, and their terminals contain CB_1_ receptors [61]. The main neurotransmitter in these nerves is acetylcholine, which influences motility, secretion and blood flow by interacting with enteric nerves. Thus, cannabinoids may also exert their antisecretory and antimotility actions at this level via the activation of presynaptic CB_1_ receptors. Currently, the presence of CB_2_ receptor markers has not been confirmed in vagal afferents. However, CB_2_ receptor-IR is present on peripheral sensory neurons and colocalizes with both CB_1_ and TRPV1 receptors, and modulate TRPV1 sensitivity via cAMP depletion [73]. If the CB_2_ receptor is also present on vagal afferents and exhibits similar colocalization, then vagal activity could be modulated by CB_2_ receptor stimulation. Stimulation of TRPV1 receptors on vagal afferents by either capsaicin or resiniferatoxin is thought to involve an initial excitatory effect which leads to neurotransmitter release (e.g. SP) in the NTS and emesis. These events are followed by desensitization and a refractory period (with possible depletion of SP in the NTS or other DVC emetic nuclei), where animals would not respond to different emetic stimuli including electrical stimulation of the vagus [70], intragastric CuSO_4_, radiation, loperamide and cisplatin in different species [74,75,76]. Indeed, immunohistochemical, molecular and electrophysiological evidence have confirmed the presence of TRPV1 receptors in the GIT vagal afferent neurons [77,78]. Thus, TRPV1 agonists such as resiniferatoxin also possess potent and broad-spectrum antiemetic activity.

### 4.3. Enteric Nervous System (ENS)

Although release of endocannabinoids in the ENS tissue has not been well investigated, the ENS appears to be an important endocannabinoid source for the GIT. Both anandamide and 2-AG can be released from non-neuronal sites such as endothelial cells [62]. Since endocannabinoids are not released from vascular smooth muscle, it is unlikely that gastrointestinal smooth muscle tissue is a source of endocannabinoids. Immunohistochemical and mRNA expression studies indicate that enzymes for the degradation of both 2-AG and anandamide (MAGL and FAAH, respectively) are present in the cell bodies and nerve fibers of myenteric neurons in the small intestine [62,63]. MAGL enzyme activity was highest in the rat duodenum and tended to decrease along the gut with lowest levels in the distal colon. Cannabinoid CB_1_ and CB_2_ receptors have distinctive distribution in the GIT, being largely concentrated in the ENS. The CB_1_ receptor is present on nerve fibers throughout the intestinal wall, but with the highest density in the two ganglionated plexi in the ENS, the myenteric and submucosal plexus [63]. Enteric ganglia consist of motor neurons, interneurons and intrinsic primary afferent neurons. Double-label immunohistochemistry indicates that CB_1_-IR colocalizes with specific markers of: (i) all cholinergic neurons (e.g. ChAT) in the guinea pig, porcine and rat myenteric plexi; (ii) most excitatory motor neurons (e.g. calretinin) to longitudinal muscles; (iii) ascending excitatory cholinergic interneurons (e.g. calretinin); (iv) some small population of SP neurons; and (v) intrinsic primary afferent neurons (e.g. calbindin) [62]. The predominant action of cannabinoids on motor neurons appears to be CB_1_ receptor-mediated presynaptic inhibition of gastrointestinal transit by attenuating transmitter release from excitatory motor neurons. Furthermore, it appears that neither CB_1_ receptors nor MAGL are colocalized with NOS-containing inhibitory neurons [62]. Thus, cannabinoid agonists are potent inhibitors of GIT contractility, and inhibition of motility from stomach to colon occurs primarily via activation of enteric CB_1_ and not CB_2_ receptors under physiological conditions [79]. This reduction in peristalsis may contribute to the peripheral antiemetic component of cannabinoid action [80]. On the other hand, in the LES, cannabinoids inhibit relaxation via the brainstem, and this effect may also in part account for their antiemetic efficacy [54,81]. 

Recent molecular and immunohistochemical evidence indicate that CB_2_ receptor mRNA and protein are also present in the majority of myenteric neurons along the GIT but not on those expressing nitric oxide synthase [82]. CB_2_ receptors do not appear to affect gut motility under normal physiological circumstances, but potentially regulate motility in pathophysiological states. In fact functional studies indicate that the CB_2_ agonist JWH133 was unable to affect the electrically-evoked twitch response of the rat ileum under physiological conditions, but inhibited this enhanced contractile response in lipopolysaccharide (LPS)-pretreated animals in a dose-dependent and CB_2_ antagonist-sensitive manner. CB_2_ receptors may also regulate tissue response to gut inflammation either by direct suppression of pro-inflammatory mediators or by affecting the response of smooth muscle to such stimuli [83]. In addition, in hyperalgesic states both CB_1_ and CB_2_ selective agonists were more potent in attenuating visceral pain produced in rodents by graded colorectal distension [83,84]. Indeed, the analgesic effects of CB_2_ receptor agonism in somatic nerve pathways have been well described, as has CB_2_-mediated inhibition of visceral nerves supplying the gastrointestinal tract [85]. TRPV1-IR has been identified in nerves within myenteric ganglia and interganglionic fiber tracts throughout the GIT. TRPV1-expressing nerves have also been observed within the: (1) muscle layers; (2) blood vessels in the gastrointestinal wall; and (3) mucosa [86,87]. In addition, TRPV1-IR is expressed by primary afferent neurons innervating the GIT. Activation of TRPV1-expressing cholinergic neurons in the myenteric plexi apparently contributes to the development of enhanced intestinal motility and secretion. Indeed, intraluminal administration of anandamide causes inflammation similar to *Clostridium difficile* toxin A in the rat ileum in a capsazepine (a TRPV1 antagonist)-sensitive manner that is not affected by cannabinoid CB_1/2_ antagonists [88]. Cholinergic secretomotor neurons also contain neuropeptide Y (NPY), while noncholinergic secretomotor nerves contain vasoactive intestinal peptide (VIP). These nerves project to the mucosa and regulate water and electrolyte levels, and are controlled through local reflexes and the CNS via sympathetic nerves. They also project to submucosal blood vessels and control blood flow. CB_1_-IR colocalizes with all VIP-containing neurons and the majority of NPY-containing neurons in the guinea-pig ileum. However, CB_1_-IR receptors do not colocalize with VIP in the porcine myenteric and submucosal plexi. Activation of CB_1_ receptors on cholinergic neurons in the submucosal plexus limits cholinergic nerve-mediated secretion, while blockade of these receptors leads to fluid accumulation in the lumen and diarrhea-like symptoms [62,84]. On the other hand, CB_2_ antagonists lack such effects.

### 4.4. Gastrointestinal Tissue

Since intestinal smooth muscle tissue does not produce endocannabinoids, intestinal tissue concentrations of 2-AG and anandamide probably reflect neuronal and nonneuronal sources such as vascular endothelial cells, intestinal epithelial cells, platelets and macrophages [61,62,63]. Large amounts of 2-AG and anadamide (44 nmol/g tissue and 36 pmol/g tissue, respectively) are present in the small intestine of mice [89,90]. In fact, mouse intestinal tissue concentration of 2-AG exceeds that of liver, spleen, lungs and kidneys by 33–55 times, and of various brain regions by 3–20 fold [89]. However, anandamide tissue levels in both the CNS and peripheral tissues can be similar, lower or greater than that present in the mouse small intestine. High intestinal levels of both 2-AG and anadamide are also present in the least shrew [91]. Distinct regional differences in endocannabinoid tissue levels appears to exist in the GIT with 2-AG being higher in the ileum than the colon and anandamide being considerably higher in the colon than the ileum, which may reflect a difference in the functional activity of these endocannabinoids in the small and large intestine. In addition, the main degradation enzymes for anandamide and 2-AG are also highly concentrated in the intestine. Stress and pathophysiologic states can affect gut endocannabinoid levels since: (1) hunger increases anandamide levels in the small intestine; (2) anandamide tissue levels increase in the rat and mouse models of colitis and in mucosal biopsy samples obtained from patients with inflammatory bowel disease; and (3) cisplatin tends to reduce 2-AG and anandamide intestinal tissue levels in least shrews [61,89,90,91]. The presence of CB_1_ receptors or its markers have been confirmed in the entire GIT on neurons supplying tissues from the stomach to the colon of several emetic and nonemetic species including humans [62,89,90,92]. However, CB_1_ receptors are differentially distributed along the length of the GIT, with the stomach and the colon being highly enriched with these receptors. Although the discussed effects of endocannabinoids on GIT motility are thought to be of neural origin, since cannabinoid CB_1_ stimulation does not directly suppress smooth muscle activity, more recent evidence indicates that the major metabolic enzyme for 2-AG degradation (MAGL), as well as CB_1_ receptors, are also highly expressed in the epithelial cells of the GIT [63,84].

## 5. Mechanisms of the Antiemetic Actions of Phyto and Synthetic Cannabinoids’ against Acute and Delayed CINV

### 5.1. Antiemetic Activity of Cannabinoid CB_1_ Receptors

Clinical studies provided the initial evidence on the antiemetic potential of Δ^9^-THC against CINV and thus preceded the customary exploratory basic mechanistic studies in animal models of vomiting [2]. The clinical trials were based both on the past anecdotal general information from Eastern cultures that cannabis products can be useful in nausea, vomiting and diarrhea, and in particular on relatively more recent reports from the 1970s that decreased emesis is often exhibited by younger patients who used marijuana while receiving chemotherapy. At least five different cannabinoids have been evaluated for their antiemetic potential in over 40 clinical trials involving phytocannabinoids (Δ^9^-THC and Δ^8^-THC) and synthetic cannabinoids (nabilone, levonantradol and nonabine) [2,93,94]. The clinical findings indicate that in general cannabinoids have a better antiemetic efficacy than dopamine D_2_ antagonist antiemetics (such as prochlorperazine, chlorpromazine, haloperidol or metchlopramide) against the frequency of vomiting episodes and severity of nausea caused by CINV. Testing of a combination of a cannabinoid agonist with a D_2_ antagonist *versus* each compound alone, has shown either no enhancement or a greater antiemetic efficacy in cancer patients receiving chemotherapy [2]. However, the dopamine D_2_ antagonists used in these early clinical trials are generally not very selective. In a recent animal study, the more selective D_2_ antagonist sulpride failed to potentiate the antiemetic efficacy of Δ^9^-THC against high-dose cisplatin-induced emesis in the least shrew model of vomiting [95]. 

Although the advent of 5-HT_3_ receptor antagonists in the 1980s led to the cessation of further cannabinoid antiemetic research in the clinic, the discovery of the cannabinoid receptors and their endogenous ligands, combined with the introduction of new animal models of emesis, have paved the way for a renaissance in the field. The first published paper providing evidence that the antiemetic effect of cannabinoids is mediated via the activation of CB_1_ (and not CB_2_) receptors was in the least shrew [4]. We envisaged that since cannabinoid CB_1_ receptor activation prevents emesis, its antagonism should cause vomiting. Indeed, large doses (10–20 mg/kg, i.p.) of SR141716A (and not the CB_2_ antagonist SR144528) produced emesis in a dose-dependent manner in least shrews and the response was blocked by both Δ^9^-THC and synthetic cannabinoids. SR141716A administration also causes nausea or emesis in 4%–14% of overweight patients who had received low doses(0.05–0.2 mg/kg) of that antagonist [96]. Likewise, SR141716A has been reported to induce vomiting in Δ^9^-THC-tolerant dogs [97]. The induced vomiting can also be attributed to the inverse agonist nature of SR141716A since other CB_1_ inverse agonists (e.g. AM251) also: (1) cause emesis [98] or potentiate the emetic efficacy of other emetogens in ferrets [6]; and (2) induce conditioned gaping in rats which is an accepted marker of nausea [99]. Overall, the discussed findings indicate that an endocannabinoid antiemetic tone may exist. Indeed, preliminary evidence indicates that unlike CB_1_ receptor inverse agonists, silent CB_1_ antagonists such as AM4113 do not induce such gastrointestinal effects [98]. Thus, the discussed antiemetic tone is probably due to a reduction in the constitutive activity of CB_1_ receptors and not via direct endogenous ligand antiemetic activity. However, SR141716A’s emetic activity is also associated with the release of large amounts of emetogenic monoamines such as DA and 5-HT in the shrew brainstem [100]. Since activation of presynaptic CB_1_ receptors inhibits neurotransmitter release [101], this could be another mechanism by which cannabinoid agonists can alleviate emesis.

### 5.2. The Broad-spectrum Antiemeic Nature of Cannabinoid CB_1_ Receptor Agonists Involves Both Central and Peripheral Emetic Loci

Δ^9^-THC and related cannabinoids (WIN55-212-2; CP55,994; HU-210) behave as broad-spectrum agonist antiemetics in a CB_1_ receptor antagonist-sensitive manner against diverse centrally- and peripherally-acting emetogens in several animal models of emesis. These emetogens include: (i) acute-phase emesis caused by cisplatin [7,66,102,103,104,105,106]; (ii) delayed-phase emesis induced by cisplatin [5,107]; (iii) the 5-HT precursor 5-hydroxytryptophan (5-HTP), the selective (e.g. 2-methylserotonin) and nonselective (e.g. 5-HT) 5-HT_3_ receptor agonists [80]; (iv) the DA precursor L-DOPA and the dopaminergic D_2_/D_3_ –receptor selective (quinpirole, quinelorane or 7-(OH) DPAT) and nonselective (apomorphine) agonists [108,109]; (v) the endocannabinoid 2-AG [11]; (vi) arachidonic acid [11]; (vii) radiation [110]; (viii) SP [111]; (ix) morphine or morphine-6-glucuronide [6,112]; (x) motion [113]; and (xi) *Staphylococcus* enterotoxin [114]. Cannabinoids’ broad-spectrum antiemetic properties against the miscellaneous central- and peripheral-acting emetogens in general, and their effectiveness against both acute- and delayed-phase CINV in animals [5] and cancer patients [107], propels this class of agonist antiemetics to the forefront of research in terms of mechanisms of action as well as sites of action. 

The central and peripheral components of antiemetic action of cannabinoids are well illustrated by our findings as follows: Low doses of Δ^9^-THC (<0.1 mg/kg, i.p.) can completely prevent the centrally-mediated 5-HT_2A_-receptor- (head-twitch and ear-scratch) behaviors produced by the brain-penetrating 5-HT_3/2A_ agonist, 2-methyl-5-HT, in a one-phase fashion in the least shrew [79]. However, Δ^9^-THC pretreatment can concomitantly attenuate the induced vomiting in a bi-phasic manner. In fact, the central emetic component of 2-methyl-5-HT was inhibited at doses less than 0.1 mg/kg, while complete abolition of the peripheral emetic component required more than 20 mg/kg Δ^9^-THC [80]. Likewise, Δ^9^-THC was four times more potent in protecting shrews from centrally-mediated 5-HTP-induced emesis in the presence of the peripheral decarboxylase inhibitor carbidopa, which prevented systemic conversion of the serotonin precursor 5-HTP to 5-HT [80]. In fact in the absence of carbidopa, 5-HTP-induced emesis was inhibited by Δ^9^-THC in a biphasic manner, while inclusion of carbidopa transformed the Δ^9^-THC-induced dose-response inhibition curve to a single central component in which Δ^9^-THC’s antiemetic efficacy was apparent at low doses. Further support for a central component of antiemetic action of cannabinoids comes from the ability of Δ^9^-THC to attenuate Fos expression induced by cisplatin in specific emetic nuclei of the DVC in the brainstem of both ferrets and shrews [5,7]. Indeed, cisplatin-induced acute vomiting causes significant Fos-IR in the NTS, DMNX and AP, whereas in the delayed phase Fos-IR was induced at relatively lower levels in the least shrew NTS and DMNX and not at all in the AP when compared to the acute phase [5]. Δ^9^-THC pretreatment not only attenuated both phases of cisplatin-induced vomiting, but also reduced the vomiting-related increases in Fos-IR during both acute and delayed phases in the shrew brainstem emetic nuclei. Furthermore, the Δ^9^-THC -induced reductions in emesis and Fos-IR were reversed by prior administration of the CB_1_ receptor antagonist, SR141716A, indicating a CB_1_ receptor antiemetic effect. Because of the differential pattern of activation of the DVC emetic nuclei during the acute and delayed phases of cisplatin-induced emesis, the discussed findings suggest that lack of activation of the AP implies that humoral signaling is unnecessary for the induction or blockade of the delayed phase vomiting [5]. However, a recent lesion study has shown that the destruction of the AP region in ferrets reduces cisplatin’s delayed phase emesis by 50% [115], while Fos expression studies indicate increased activity in the rat AP during a 48 h cisplatin exposure [116]. Since under physiological conditions 5-HT exists mainly in the ionized form, it is generally accepted that unlike its precursor (5-HTP), serotonin cannot penetrate the BBB to induce vomiting [1,13]. Instead, as discussed earlier, systemically- administered serotonin is thought to induce emesis via activation of 5-HT_3_ receptors present on the vagal afferents [13,30]. In fact, intraperitoneal administration of the relatively selective and brain-penetrating 5-HT_3_ receptor agonist, 2-methyl-5-HT, in the least shrew causes emesis as well as increasing the vomiting-related Fos-IR in both the brainstem emetic nuclei (AP, DMNX and NTS) and in the enteric nervous system in the GIT [5]. Indeed, Δ^9^-THC can prevent peripherally-mediated serotonin-induced emesis, but only at high doses and via a single component [80]. 

Results from a single-dose combination study indicate that the antiemetic efficacy of ondansetron(a 5-HT_3_ antagonist) plus dexamethasone was not potentiated by Δ^9^-THC in patients receiving chemotherapy [117]. However, dose-response studies do indicate that low doses of either ondansetron or tropisetron can potentiate the antiemetic efficacy of low but not high doses of Δ^9^-THC against cisplatin-induced emesis in both the least and house musk shrews [105,118]. Although generally disappointing, the lack of persistent additive or synergistic antiemetic action across doses when a cannabinoid agonist is combined with a 5-HT_3_ antagonist, is not surprising. There is likely to be a large overlap in the mechanisms by which these drugs block emesis, which would prevent the hoped-for enhanced antiemetic effect. For example, the mechanism of CB_1_ receptor antiemetic agonists, as stated above, likely relies on presynaptic inhibition. This CB_1_-mediated inhibition (e.g. in the DVC or GI nerve plexi) could reduce antiemetic activity generated by postsynaptic, tropisetron-sensitive, 5-HT_3_ receptor-containing neurons, or by presynaptic terminals which might colocalize these 5-HT_3_ receptors [119]. In fact, there is also evidence that cannabinoids can directly modulate 5-HT_3_ receptors allosterically [120,121]. If this direct crosstalk is also part of the mechanism of cannabinoid-mediated antiemesis, any potential additive effect may be dampened by interference from 5-HT_3_ antagonist binding. The slight enhancement of antiemetic ability by low doses of Δ^9^-THC in combination with low doses of tropisetron would result from incomplete receptor occupancy by either or both drugs, or possibly by incomplete anatomical overlap of cannabinoid and 5-HT_3_ receptors. 

Dopamine may also induce emesis via central as well as peripheral anatomical substrates of vomiting [1]. Using a similar logic to that which explains the biphasic nature of 5-HT inhibition, diverse cannabinoids seem to prevent emesis caused either by the DA precursor L-DOPA (with or without carbidopa), or by the brain-penetrating direct-acting D_2_/D_3_ selective agonists, through a single component inhibition curve which may indicate the importance of a solitary site of antiemetic action of cannabinoids against DA-induced emesis [108,109]. However, this requires further confirmation. 

Δ^9^-THC also inhibits the ability of another identified emetogenic transmitter of CINV, SP, in a dose-dependent manner in the least shrew [111]. Unlike the well-accepted dogma that SP is mainly involved during the delayed CINV phase, both recent studies in the least shrew brainstem and jejunum [16], as well as clinical data in cancer patient’s plasma [20,122], have shown that large amounts of this peptide are released during both phases of cisplatin-induced vomiting. Moreover, SP-induced vomiting involves both central [123] and peripheral [40] mechanisms. Furthermore, as already discussed, Δ^9^-THC not only inhibits SP-induced emesis in a dose-dependent manner via CB_1_ receptors, but also blocks both the immediate and delayed phases of emesis caused by cisplatin [5,107]. 

Finally, addition of the anti-inflammatory glucocorticoid dexamethasone seems to add to the antiemetic potential of cannabinoids in cancer patients receiving chemotherapy [124]. However, a recent multi dose-response combination study against high-dose cisplatin in the least shrew failed to show a dose-dependent interaction during the acute phase CINV [118]. In the case of dexamethasone, effects on emetic behavior would be mediated “downstream” from the presynaptic events modulated by CB_1_ receptors. Postsynaptic second-messenger systems, including the prostanoid-producing arachidonic acid metabolic pathways, would provide an interface through which dexamethasone- and cannabinoid-mediated systems would overlap. The net effect in this case would be cannabinoid-mediated inhibition, or lack of stimulation, of neurons whose downstream antiemetic effecter mechanisms were already inhibited by dexamethasone, preventing the proposed enhancement of antiemetic activity by the combined drug regimen.

## 6. Mechanisms via Which Cannabinoids Prevent Chemotherapy-Induced Acute and Delayed Phase Emesis

Antiemetic therapy has become integral to management of cancer patients. Research in the past 25 years has led to improvements in the control of CINV, by which 75%–80% of patients can be protected via the use of antiemetic cocktails. Cisplatin exposure produces vomiting biphasically in both humans [20] and other vomiting specis [15,16,17,18,19]. In patients, the acute (immediate) emetic phase is comprised of episodes occurring within 24 hours of cisplatin exposure and the delayed phase between days 2–7 post-infusion. The current antiemetic therapy dogma is based upon the premise that during acute vomiting cisplatin induces 5-HT release from EC cells, which stimulates local 5-HT_3_ receptors on gastrointestinal vagal afferents to initiate the vomiting reflex [30]. The delayed phase emesis is thought to be due to activation of brainstem tachykininergic NK_1_ receptors subsequent to the release of SP in the DVC [12]. Based on the latter hypothesis, the current antiemetic regimens include a 5-HT_3_ receptor antagonist (e.g. ondansetron) plus a corticosteroid (such as dexamethasone) for the prevention of the acute vomiting and an NK_1_ receptor antagonist (e.g. aprepitant) for the delayed emesis. Although these findings are important breakthroughs in oncology, the incidence of nausea and vomiting still remains unacceptably high, and is a major factor in premature discontinuation of chemotherapy. Moreover, the discussed CINV-based neurotransmitter hypothesis is too simplistic. Indeed, it is mainly focused on one neurotransmitter in isolation per emetic phase via a well established mechanism in either the GIT or brainstem respectively. Furthermore, it excludes interactions not only between emetic neurotransmitters at each peripheral and CNS emetic locus, but also between brain-gut emetic circuits [1]. Our inability to develop more effective antiemetic regimens against CINV is due to having only a partial appreciation of relative temporal and spatial contributions of multiple emetic neurotransmitters (DA, 5-HT, SP, eicosanoids such as prostaglandins, leukotrienes and endocannabinoids as well as related downstream emetic metabolites) which have differential and overlapping sequential release and interplay in the regulation of both phases of CINV, and in both the brainstem and the GIT. Consequently, we recently challenged the neurotransmitter and anatomical bases of the established dogma in favor of a hypothesis that proposes multiple but differential and overlapping release of several neurotransmitters (DA, 5-HT, SP, prostaglandins and related emetic substances) during each phase of CINV [1]. Clinical evidence is supportive of this notion since no single antiemetic is completely effective at blocking emesis in either phase, but when administered together, the antiemetic efficacy of the combination is greater than that of each agent given individually [20]. The already described broad-spectrum antiemetic efficacy of Δ^9^-THC against diverse peripherally- and/or centrally-acting emetogens (such as serotonin, dopamine, substance P and prostaglandins); and the discussed nature of Δ^9^-THC’s central and peripheral components of antiemetic actions against such emetogens (see section 5.1–5.2); provide both the neurotransmitter and neuroanatomical bases for cannabinoids to attenuate the immediate and delayed phases of CINV in animals [5] and humans [107]. 

## 7. The Nature of Pro- and Antiemetic Actions of Endocannabinoids and Endovanilloids

The antiemetic efficacy of phyto- and synthetic cannabinoid agonists and the emetogenic potential of CB_1_ receptor antagonists led us to hypothesize that endocannabinoids should attenuate cisplatin-induced vomiting. However, exogenous administration of either anandamide or 2-AG in the least shrew lacked efficacy against cisplatin’s vomiting (Darmani, unpublished findings). On the other hand, cisplatin caused dose- and time-dependent increases in endogenous basal levels of 2-AG but not anandamide in the least shrew brain, while concomitantly reducing intestinal tissue concentrations of both endocannabinoids [125]. Moreover, intraperitoneal injection of 2-AG was shown to cause dose-dependent emesis at low doses (1–2.5 mg/kg, i.p.) in a CB_1_ antagonist-sensitive manner, whereas anandamide was emetogenic at 10 mg/kg but not at lower or higher doses, while its more stable analog methanandamide lacked emetic activity [11]. We have attributed the emetogenicity of 2-AG to its rapid metabolism since its major metabolite (arachidonic acid) is also a potent vomit inducer, and the emetic capacity of both emetogens can be prevented in the least shrew by the cyclooxygenase inhibitor, indomethacin. Not surprisingly, indomethacin has also been shown to attenuate cisplatin-induced emesis in piglets [126]. Furthermore, pretreatment with either anandamide, methanandamide, phyto-, or synthetic cannabinoids prevents the ability of 2-AG to cause emesis in the least shrew [11]. In addition, some downstream metabolites of arachidonic acid such as PGE_2_ and PGF_2__α_ that are products of cyclooxygenase enzymes are emetogenic in several species including humans [127], piglets [128], and least shrews [125]. Moreover, the rise in plasma concentrations of the cited prostaglandins, some leukotrienes or 5-HETE are associated with vomiting under some conditions, including pregnancy in humans [129] and *S. aureus* enterotoxin B exposure in monkeys [130]. In fact, systemic administration of leukotriene LTC_4_ in the least shrew not only causes vomiting but also results in Fos expression in the emetic nuclei of the DVC and in the ENS [60].

Indirect agonists of the endocannabinoid system such as selective inhibitors of FAAH (AA-5-HT or URB597) or selective reuptake inhibitors (OMDM1 or VDM11) , have also been tested in the least shrew against several emetogens (cisplatin, apomorphine, or 2-AG) but none of them had consistent antiemetic activity [11]. In fact, some of these (AA-5-HT, URB597 and OMDM1) at larger doses (> 10 mg/kg, i.p.) caused emesis by themselves in least shrews. There appears to be some species differences in the emetic/antiemetic efficacy of endocannabinoids and their indirect agonists. For example, in the ferret methanandamide causes retching but not vomiting [6], while anandamide, 2-AG, VDM11, and URB5973 lacked emetic/retching activity at 2–3 mg/kg doses. The inability of the ferret to vomit in response to intraperitoneal injection of 2-AG may not be surprising, since neither 5-HT nor SP can induce emesis in this species via the peripheral routes [1]. Furthermore, the discussed lower doses of these compounds appear to prevent vomiting caused by morphine-6-glucuronide in the ferret via activation of both CB_1_ and CB_2_ receptors [59]. However, previous reports from the latter authors as well as numerous other publications have discounted a direct role for CB_2_ receptors in emesis. Overall, the discussed findings suggest that, depending on the dose utilized, endocannabinoids and indirect-acting cannabinoid agonists may possess mixed emetic/antiemetic activity.

Not only is anandamide an endocannabinoid, it also behaves as an endovanilloid and may produce its antiemetic activity via stimulation of both cannabinoid CB_1_ and vanilloid TRPV1 receptors. Indeed, antiemetic actions of anandamide and other hybrid compounds such as arvanil and NADA against morphine-6-glucuronide-induced vomiting can be reversed in ferrets by either CB_1_ or TRPV1 antagonist pretreatment [9]. In fact potent and selective agonists of TRPV1 receptors such as resiniferatoxin exhibit an initial emetic activity by themselves, and subsequently show antiemetic efficacy when tested against a diverse array of emetogens [1]. Gastrointestinal resiniferatoxin-sensitive vagal afferent C-fiber terminals contain SP, as well as TRPV1 receptors, and stimulation of these receptors seems to release SP to activate neurons of the mNTS [1]. These neurons in turn drive the CPG to induce vomiting. However, the enhanced firing in the mNTS gradually subsides and the response of these neurons to stimulation of abdominal afferents disappears due to desensitization simultaneously with the cessation of vomiting. This probably in part accounts for the broad-spectrum nature of the antiemetic efficacy of resiniferatoxin. Another factor contributing towards the broad antiemetic clinical potential of potent synthetic hybrid antiemetics is the concomitant stimulation of antiemetic CB_1_ and TRPV1 receptors. In fact both receptor classes are distributed in a similar pattern in the neurons of the emetic nuclei of the DVC and are colocalized in the mNTS, in motor neurons of the DMNX, and in a few scattered neurons of the AP [9]. These findings further add to the broad-spectrum antiemetic nature of cannabinoids and vanilloids against both phases of CINV [5,76,107]. The antiemetic locus of CB_1_ and TRPV1 receptor activity probably lies both in the vagal afferent/efferent neurons and NTS [1]. More recent multilabeling evidence also indicates that not only does CB_1_ colocalize with punctate immunoreactivity for 5-HT or SP neuronal terminals in the NTS, but on some puncta at this locus, colocalization of all three antigens is present [5]. Activation of CB_1_ receptors may also oppose the emetogenic effects of both 5-HT and SP at the level of the vagus and myenteric plexus (see sections 4.5 and 5.5). Like resiniferatoxin, anandamide can cause emesis in a non-dose-dependent manner [11]. However, among the emetic agents tested, anandamide provided protection against 2-AG and morphine-6-glucuronide, but not cisplatin [11,59], and Darmani, unpublished observations.

## 8. Maijuana and Hyperemesis Syndrome

With the rising interest in cannabinoid use in therapeutics, the safety of cannabinoids is an emerging source of concern for many clinicians. Serious adverse events reported in randomized controlled trials of medical cannabinoid preparations invovle the respiratory (e.g. dyspnea, pneumonia), gastrointestinal (vomiting, diarrhea) and nervous system (e.g. dizziness, acute panic) [131]. Cannabis-induced hyperemesis is a recently recognized syndrome associated with chronic cannabis use [132]. It is characterized by repeated cyclical vomiting and learned compulsive hot water bathing behavior. Although considered rare, recent international publications of numerous case reports suggest the contrary. The syndrome appears to be a paradox and the pathophysiological mechanism(s) underlying the induced vomiting remain unknown. Although some traditional hypotheses have already been proposed, a recent review contained elsewhere in this volume critically explores the basic science mechanisms which may underly the induced vomiting and the associated learned hot bathing behavior for temporary relief from the hyperemesis [133]. These encompass: (1) pharmacokinetic factors such as long half-life, chronic exposure, lipid solubility, individual variation in metabolism/ excretion leading to accumulation of emetogenic cannabinoid metabolites, and/or cannabinoid withdrawal; and (2) pharmacodynamic factors including switching of the efficacy of Δ^9^-THC from partial agonist to antagonist; differential interaction of Δ^9^-THC with Gs and Gi signal transduction proteins; CB_1_ receptor desensitization or downregulation, alterations in tissue concentrations of endocannabinoid agonists/inverse agonists; Δ^9^-THC-induced mobilization of emetogenic metabolites of the arachidonic acid cascade; brainstem *versus* enteric actions of Δ^9^-THC, and/or hypothermic *versus* hyperthermic actions of Δ^9^-THC.
